# Development and validation of a machine learning-based early warning model for bone metastasis in newly diagnosed prostate cancer

**DOI:** 10.3389/fonc.2026.1815465

**Published:** 2026-07-09

**Authors:** Leibo Wang, Wei He, Changyong Zhao, Qi lv, Tao Qiu, Jianpo Zhai, Kaiyi Mao, Daobing Li, Xian Wen

**Affiliations:** 1 Department of Urology, Affiliated Hospital of Zunyi Medical University, Zunyi, China; 2 Surgery, Beijing Jishuitan Hospital Guizhou Hospital, Guiyang, China; 3 Department of Urology, Beijing Jishuitan Hospital, Beijing, China; 4 Shenzhen Nanshan Medical Group Headquarter, Shenzhen, China

**Keywords:** bone metastasis, machine learning, prostate cancer, PSA, Shap

## Abstract

**Background:**

Bone metastasis (BM) is common in newly diagnosed prostate cancer (PCa), particularly in patients with advanced disease at presentation. However, the indications for bone scintigraphy remain inconsistent and may lead to unnecessary imaging in low-risk patients. This study aimed to develop and validate a machine learning model for individualized prediction of BM in patients with newly diagnosed PCa.

**Methods:**

We retrospectively collected data from 327 patients with newly diagnosed PCa from two tertiary hospitals. Patients were randomly assigned to a training set (n = 229) and an internal validation set (n = 98). The Boruta algorithm was used to identify significant predictors. Seven machine learning models, including random forest and logistic regression, were developed and evaluated using five-fold cross-validation. Model performance was evaluated using receiver operating characteristic (ROC) curves, calibration, and decision curve analysis (DCA). The best-performing model was interpreted using SHapley Additive exPlanations (SHAP) and deployed as an online prediction tool.

**Results:**

Six predictors were identified by the Boruta algorithm: clinical T stage, Gleason score, total prostate-specific antigen (tPSA), alkaline phosphatase (ALP), regional lymph node metastasis, and fibrinogen. Among the seven models, the random forest model achieved the best performance, with an area under the curve (AUC) of 0.902 in the training set and 0.906 in the internal validation set. Calibration curves showed good agreement between predicted and observed outcomes, and decision curve analysis indicated favorable clinical utility. An interactive online prediction tool was developed for individualized risk estimation.

**Conclusion:**

We developed and internally validated an interpretable random forest model for predicting BM in newly diagnosed PCa. This model may help identify high-risk patients and guide the use of bone scintigraphy. Prospective multicenter studies with external validation are needed to further confirm its generalizability.

## 1 Introduction

Prostate cancer is the second most commonly diagnosed malignancy among men worldwide and the fifth leading cause of cancer-related death globally, with approximately 1.6 million new cases and 366,000 deaths reported annually (1). Notably, studies have shown that the proportion of Chinese patients with bone metastases at the time of initial diagnosis ranges from 13.3% to 26%, significantly higher than that in European countries (3%–10%) and the United States (3%–5%) (2). The pelvis and spine are the most frequently affected sites. Bone metastases(BM) not only cause skeletal-related events such as bone pain, limited mobility, and increased risk of fractures, but also deprive patients of the opportunity for curative surgical treatment, making them a leading cause of mortality in PCa patients (3). Therefore, early identification of bone metastases is of great clinical importance for improving patient outcomes.

Currently, bone scintigraphy using technetium-99m methylene diphosphonate (99mTc-MDP) is the most commonly employed imaging modality for detecting PCa bone metastases. While it offers high sensitivity for metastatic lesions, its specificity is relatively low (2). Indications for bone scintigraphy vary across guidelines. According to the European Association of Urology (EAU) (4), bone scans are recommended only in patients with a total prostate-specific antigen(tPSA) level ≥20 ng/mL and poorly differentiated tumors. In contrast, the Chinese Guidelines for the Diagnosis and Treatment of Prostate Cancer recommend 99mTc-MDP bone scans for all newly diagnosed PCa patients (5). However, routine bone scanning may lead to unnecessary radiation exposure and increased healthcare costs, and its universal application in all PCa patients at initial diagnosis remains controversial.

With advances in computational technology, machine learning(ML) has emerged as a powerful tool in medical research, capable of analyzing large-scale, heterogeneous, and complex clinical datasets to develop predictive models that can support decision-making in cancer diagnosis and management (6, 7).

Accordingly, this study aimed to develop and validate a machine learning model for predicting bone metastasis in newly diagnosed PCa. We further applied SHapley Additive exPlanations (SHAP) for model interpretation and deployed the optimal model as a web-based tool, with the ultimate goal of facilitating early, individualized risk stratification and clinical management of prostate cancer bone metastases.

## 2 Materials and methods

### 2.1 Study population and design

This retrospective study included 327 patients with newly diagnosed prostate cancer from two centers: 276 patients from Zunyi Medical University Affiliated Hospital (January 1, 2019, to December 30, 2023) and 51 patients from Beijing Jishuitan Hospital (January 1, 2020, to December 30, 2022). Inclusion criteria were as follows: (1) Histopathological confirmation of prostate cancer obtained via ultrasound-guided prostate biopsy or surgery. (2) Clear diagnosis of bone metastasis based on whole-body bone scintigraphy or other imaging modalities during hospitalization. Bone metastasis was primarily diagnosed using whole-body bone scintigraphy with ^99m^Tc MDP. A positive scan was defined as two or more focal areas of increased tracer uptake with a distribution pattern suggestive of metastasis (e.g., spine, pelvis, ribs). For patients with solitary or equivocal lesions, confirmatory imaging included MRI for spine/pelvis, CT for other skeletal sites, or ^18^F NaF PET/CT when available. All scans were independently reviewed by two experienced nuclear medicine physicians. Persistent equivocal findings were resolved by follow-up imaging at 3–6 months or histopathological biopsy when clinically feasible. Histopathological confirmation was not routinely obtained due to invasiveness, but performed in diagnostically challenging cases. These criteria reflect current clinical practice and provide a robust reference standard for BM diagnosis. (3) Complete clinical and radiological data available. Exclusion criteria included: (1) History of prostate surgery, radiotherapy, chemotherapy, hormonal therapy, or targeted therapy. (2) Presence of other malignancies that could confound the identification of bone metastases. (3) Coexisting bone injuries or known bone metabolic diseases. (4) Incomplete medical records. Patients were randomly divided into a training set (n = 229) and an internal validation set (n = 98) at a ratio of 7:3. This study was approved by the Ethics Committee of Guizhou Hospital, Beijing Jishuitan Hospital (20220402) and the Ethics Committee of Affiliated Hospital of Zunyi Medical University. All patient data were anonymized, and the online prediction tool does not collect or store any identifiable information. Given the retrospective design and minimal risk to participants, informed consent was waived. All patient data were anonymized by removing direct identifiers before analysis.

### 2.2 Clinical data collection

Clinical variables collected included: age, histological subtype, Gleason score, clinical T-stage (cT), regional lymph node metastasis, total prostate-specific antigen, tPSA/free PSA ratio (t/fPSA), alkaline phosphatase, serum calcium, phosphorus, albumin(Alb), prealbumin, albumin-to-globulin ratio (AGR), absolute neutrophil count, absolute lymphocyte count, platelet count, fibrinogen, hemoglobin (Hb), mean corpuscular volume (MCV), systemic immune-inflammation index (SII), prognostic nutritional index (PNI), neutrophil-to-lymphocyte ratio (NLR), and platelet-to-lymphocyte ratio (PLR). The calculated indices were defined as follows: SII = platelet count × neutrophil count/lymphocyte count (10^9^/L). PNI = serum albumin (g/L) + 5 × absolute lymphocyte count (10^9^/L). NLR = neutrophil count/lymphocyte count (10^9^/L). PLR = platelet count/lymphocyte count (10^9^/L).

### 2.3 Data preprocessing and feature selection

Before analysis, variables with more than 20% missing values were excluded. No variable in the current dataset exceeded this threshold. For the remaining variables (missing rate <5%, primarily in fibrinogen and ALP), missing continuous values were imputed using multivariate imputation by chained equations (MICE) with five iterations, and missing categorical values were imputed using the mode of the training set. All continuous features were then standardized using Z score normalization (mean = 0, SD = 1) based solely on the training set. Categorical variables (clinical T stage, Gleason score group, regional lymph node metastasis, ALP category, tPSA category) were encoded using treatment contrasts (dummy coding). To prevent data leakage, all preprocessing steps including imputation, normalization, and encoding were performed exclusively on the training set, and the same parameters (e.g., imputed values, means, standard deviations, dummy coding schemes) were applied to the internal validation set. Because the prevalence of bone metastasis in the training set was 52.4% (120/229), no additional class balancing technique (e.g., SMOTE) was applied. Feature selection was performed using the Boruta algorithm.

### 2.4 Statistical analysis

All statistical analyses were performed using R software (version 4.3.3). Continuous variables with normal distribution were expressed as mean ± standard deviation and compared using Student’s t-test. Non-normally distributed variables were reported as median (interquartile range) and compared using the Mann–Whitney U test. Categorical variables were compared using the Chi-square test. The Boruta algorithm was applied to the training set to identify relevant predictors of bone metastasis in newly diagnosed PCa. The cutoff values for tPSA (<20, 20–100, ≥100 ng/mL) and Gleason score (<8, 8, >8) were based on established clinical guidelines (4, 8), and the ALP cutoff (≤125, >125 U/L) was derived from prior literature (2) and institutional reference ranges. Based on selected features, seven machine learning models were constructed: Artificial neural network (ANN), Decision tree (DT), Logistic regression (LR), Random forest (RF), Support vector machine (SVM), Naive Bayes classifier, eXtreme Gradient Boosting (XGBoost). Model hyperparameters were optimized using grid search with five-fold cross-validation. Five-fold cross-validation was used to evaluate the generalizability of each model. Model performance was assessed using the following metrics: Area under the receiver operating characteristic curve, Accuracy, Precision, Recall, Specificity, F1 Score. The best-performing model underwent interpretability analysis using SHAP. SHAP values quantified each feature’s contribution to the model’s prediction and provided local explanations for individual predictions, revealing how each feature influenced the outcome on a case-by-case basis.

Finally, the optimal model was used to develop a web-based application for personalized risk prediction of bone metastasis in newly diagnosed PCa. Calibration curves were used to assess the agreement between predicted and observed probabilities, and decision curve analysis was employed to evaluate the clinical utility and net benefit of the predictive model.

## 3 Results

### 3.1 Clinical data of the training and validation groups

This study included 327 patients with prostate cancer: 276 from Zunyi Medical University Affiliated Hospital and 51 from Beijing Jishuitan Hospital. Patients were randomly divided into a training set (n = 229) and a validation set (n = 98) in a 7:3 ratio. Baseline characteristics were well balanced between the two cohorts (all *P* > 0.05) (**Table 1**). In the training set, 120 patients (52.40%) had bone metastasis.

**Table 1:** Comparison of clinical features between training set and validation set.

Variables	Total (n = 327)	Validation (n = 98)	Train (n = 229)	*P*
Status, n(%)				0.654
non-BM	153 (46.79)	44 (44.90)	109 (47.60)	
BM	174 (53.21)	54 (55.10)	120 (52.40)	
Alb, Mean ± SD	37.38 ± 4.34	37.32 ± 4.31	37.50 ± 4.43	0.731
Prealbumin, Mean ± SD	215.35 ± 61.12	214.93 ± 61.53	216.31 ± 60.45	0.853
t/fPSA, M (Q_1_, Q_3_)	0.12 (0.08, 0.18)	0.12 (0.08, 0.17)	0.13 (0.08, 0.23)	0.129
Ca, M (Q_1_, Q_3_),mg	2.22 (2.13, 2.30)	2.23 (2.14, 2.30)	2.22 (2.12, 2.31)	0.902
P, M (Q_1_, Q_3_), mmol/L	1.03 (0.92, 1.14)	1.03 (0.91, 1.14)	1.03 (0.93, 1.11)	0.738
AGR, M (Q_1_, Q_3_)	1.45 (1.29, 1.62)	1.43 (1.28, 1.61)	1.50 (1.30, 1.64)	0.532
Hb, M (Q_1_, Q_3_), g/L	128.00 (117.00, 142.50)	128.00 (117.00, 143.00)	128.00 (117.00, 141.75)	0.904
MCV, M (Q_1_, Q_3_)	92.20 (89.40, 95.60)	92.20 (89.50, 95.30)	92.00 (89.40, 96.25)	0.787
Absolute lmphocyte count, M(Q_1_, Q_3_)	1.35 (1.02, 1.79)	1.33 (1.01, 1.74)	1.45 (1.08, 1.85)	0.171
Absolute neutrophil count, M (Q_1_, Q_3_)	3.77 (2.86, 4.97)	3.72 (2.90, 4.99)	3.84 (2.69, 4.93)	0.376
Platelet count, M(Q_1_, Q_3_)	194.00 (157.50, 240.00)	194.00 (162.00, 240.00)	196.50 (149.25, 239.75)	0.590
Fibrinogen, M (Q_1_, Q_3_), g/L	3.37 (2.69, 4.39)	3.44 (2.65, 4.39)	3.28 (2.71, 4.41)	0.499
SII, M (Q_1_, Q_3_)	525.08 (336.01, 900.32)	533.63 (358.80, 903.14)	499.92 (292.10, 869.85)	0.173
PNI, M (Q_1_, Q_3_)	44.35 (40.80, 48.88)	44.15 (40.55, 48.35)	44.83 (41.30, 49.08)	0.348
NLR, M (Q_1_, Q_3_)	2.72 (1.90, 4.10)	2.79 (2.04, 4.10)	2.58 (1.76, 4.05)	0.197
PLR, M (Q_1_, Q_3_)	138.22 (106.07, 196.85)	144.85 (110.00, 197.45)	131.01 (100.40, 194.64)	0.177
Age, n(%)				0.303
<70year	151 (46.18)	110 (48.03)	41 (41.84)	
≥70year	176 (53.82)	119 (51.97)	57 (58.16)	
ALP, n(%)				0.650
<125u/L	238 (72.78)	165 (72.05)	73 (74.49)	
≥125u/L	89 (27.22)	64 (27.95)	25 (25.51)	
Pathology, n(%)				0.168
Adenocarcinoma	309 (94.50)	219 (95.63)	90 (91.84)	
Non-adenocarcinoma	18 (5.50)	10 (4.37)	8 (8.16)	
T, n(%)				0.303
T1/T2	146 (44.65)	98 (42.79)	48 (48.98)	
T3/T4	181 (55.35)	131 (57.21)	50 (51.02)	
N, n(%)				0.781
NO	184 (56.27)	130 (56.77)	54 (55.10)	
YES	143 (43.73)	99 (43.23)	44 (44.90)	
Gleason score, n(%)				0.735
<8	47 (14.37)	32 (13.97)	15 (15.31)	
8	100 (30.58)	73 (31.88)	27 (27.55)	
>8	180 (55.05)	124 (54.15)	56 (57.14)	
tPSA, n(%)				0.447
<20ng/ml	66 (20.18)	47 (20.52)	19 (19.39)	
20-100ng/ml	104 (31.80)	68 (29.69)	36 (36.73)	
≥ 100ng/ml	157 (48.01)	114 (49.78)	43 (43.88)	

The clinical and laboratory characteristics stratified by bone metastasis status in the training set are shown in **Table 2**. Compared with the non-BM group, patients with bone metastasis had significantly lower levels of albumin, hemoglobin, AGR, and PNI, but higher levels of fibrinogen, SII, and PLR (all *P* < 0.05). Elevated ALP, advanced T stage, lymph node metastasis, higher Gleason score, and higher tPSA levels were more common in patients with bone metastasis (all *P* < 0.001). No significant differences were observed in age, pathological type, prealbumin, t/fPSA ratio, MCV, neutrophil count, platelet count, lymphocyte count, or NLR (*P* > 0.05).

**Table 2:** Comparison of clinical characteristics between BM and non-BM patients in the training set.

Variables	Total (n = 229)	non-BM (n = 109)	BM (n = 120)	*P*
Alb, Mean ± SD	37.32 ± 4.31	38.21 ± 4.02	36.52 ± 4.42	0.003
Prealbumin, Mean ± SD	214.93 ± 61.53	219.82 ± 52.03	210.50 ± 68.96	0.247
t/fPSA, M (Q_1_, Q_3_)	0.12 (0.08, 0.17)	0.11 (0.07, 0.17)	0.12 (0.08, 0.18)	0.224
Ca, M (Q_1_, Q_3_),mg	2.23 (2.14, 2.30)	2.24 (2.17, 2.30)	2.20 (2.12, 2.29)	0.015
P, M (Q_1_, Q_3_), mmol/L	1.03 (0.91, 1.14)	1.00 (0.91, 1.10)	1.06 (0.92, 1.17)	0.040
AGR, M (Q_1_, Q_3_)	1.43 (1.28, 1.61)	1.50 (1.32, 1.67)	1.40 (1.22, 1.60)	0.003
Hb, M (Q_1_, Q_3_), g/L	128.00 (117.00, 143.00)	135.00 (122.00, 146.00)	125.00 (108.25, 137.25)	<.001
MCV, M (Q_1_, Q_3_)	92.20 (89.50, 95.30)	93.20 (90.10, 95.30)	91.80 (89.20, 94.95)	0.147
Absolute lmphocyte count, M (Q_1_, Q_3_)	1.33 (1.01, 1.74)	1.43 (1.02, 1.87)	1.26 (0.98, 1.68)	0.074
Absolute neutrophil count, M (Q_1_, Q_3_)	3.72 (2.90, 4.99)	3.60 (2.85, 4.86)	3.83 (2.92, 5.29)	0.640
Platelet count, M (Q_1_, Q_3_)	194.00 (162.00, 240.00)	184.00 (160.00, 228.00)	197.50 (167.00, 249.25)	0.067
Fibrinogen, M (Q_1_, Q_3_),g/L	3.44 (2.65, 4.39)	3.09 (2.52, 3.72)	3.82 (3.00, 4.77)	<.001
SII, M (Q_1_, Q_3_)	533.63 (358.80, 903.14)	498.41 (330.38, 773.45)	626.17 (419.62, 1004.80)	0.025
PNI, M (Q_1_, Q_3_)	44.15 (40.55, 48.35)	45.70 (42.30, 49.00)	43.20 (39.14, 47.39)	0.002
NLR, M (Q_1_, Q_3_)	2.79 (2.04, 4.10)	2.64 (1.93, 3.73)	2.89 (2.09, 4.40)	0.252
PLR, M (Q_1_, Q_3_)	144.85 (110.00, 197.45)	129.70 (100.00, 179.79)	164.78 (122.54, 217.13)	0.002
Age, n(%)				0.865
<70year	110 (48.03)	53 (48.62)	57 (47.50)	
≥70year	119 (51.97)	56 (51.38)	63 (52.50)	
ALP, n(%)				<.001
≤125u/L	165 (72.05)	104 (95.41)	61 (50.83)	
>125u/L	64 (27.95)	5 (4.59)	59 (49.17)	
Pathology, n(%)				0.866
Adenocarcinoma	219 (95.63)	105 (96.33)	114 (95.00)	
Non-adenocarcinoma	10 (4.37)	4 (3.67)	6 (5.00)	
T, n(%)				<.001
T1/T2	98 (42.79)	68 (62.39)	30 (25.00)	
T3/T4	131 (57.21)	41 (37.61)	90 (75.00)	
N, n(%)				<.001
NO	130 (56.77)	89 (81.65)	41 (34.17)	
YES	99 (43.23)	20 (18.35)	79 (65.83)	
Gleason, n(%)				<.001
<8	32 (13.97)	29 (26.61)	3 (2.50)	
8	73 (31.88)	39 (35.78)	34 (28.33)	
>8	124 (54.15)	41 (37.61)	83 (69.17)	
tPSA, n(%)				<.001
<20ng/ml	47 (20.52)	44 (40.37)	3 (2.50)	
20-100ng/ml	68 (29.69)	43 (39.45)	25 (20.83)	
≥100ng/ml	114 (49.78)	22 (20.18)	92 (76.67)	

### 3.2 Selection of predictive factors for prostate cancer bone metastasis risk

**Figure 1:**
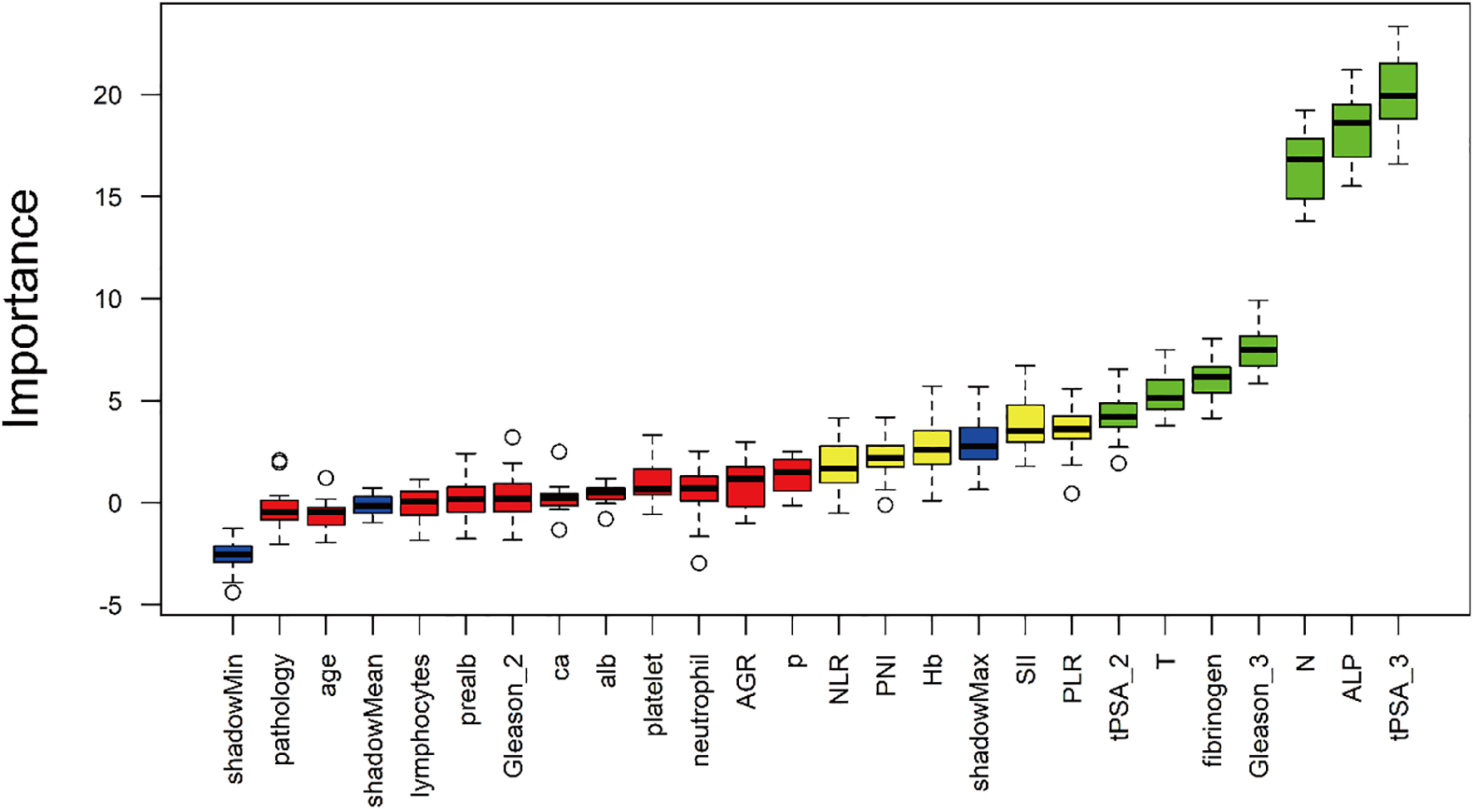
Boruta feature selection results. Green boxes represent confirmed important features, yellow boxes represent tentative features, red boxes represent rejected features, and blue boxes represent shadow features used as references by the Boruta algorithm.

The Boruta algorithm identified six variables as predictive factors for prostate cancer bone metastasis: tPSA, ALP > 125 U/L, regional lymph node metastasis, Gleason score > 8 (Gleason_3), T stage, and fibrinogen (**Figure 1**).

### 3.3 Evaluation of machine learning models

**Figure 2:**
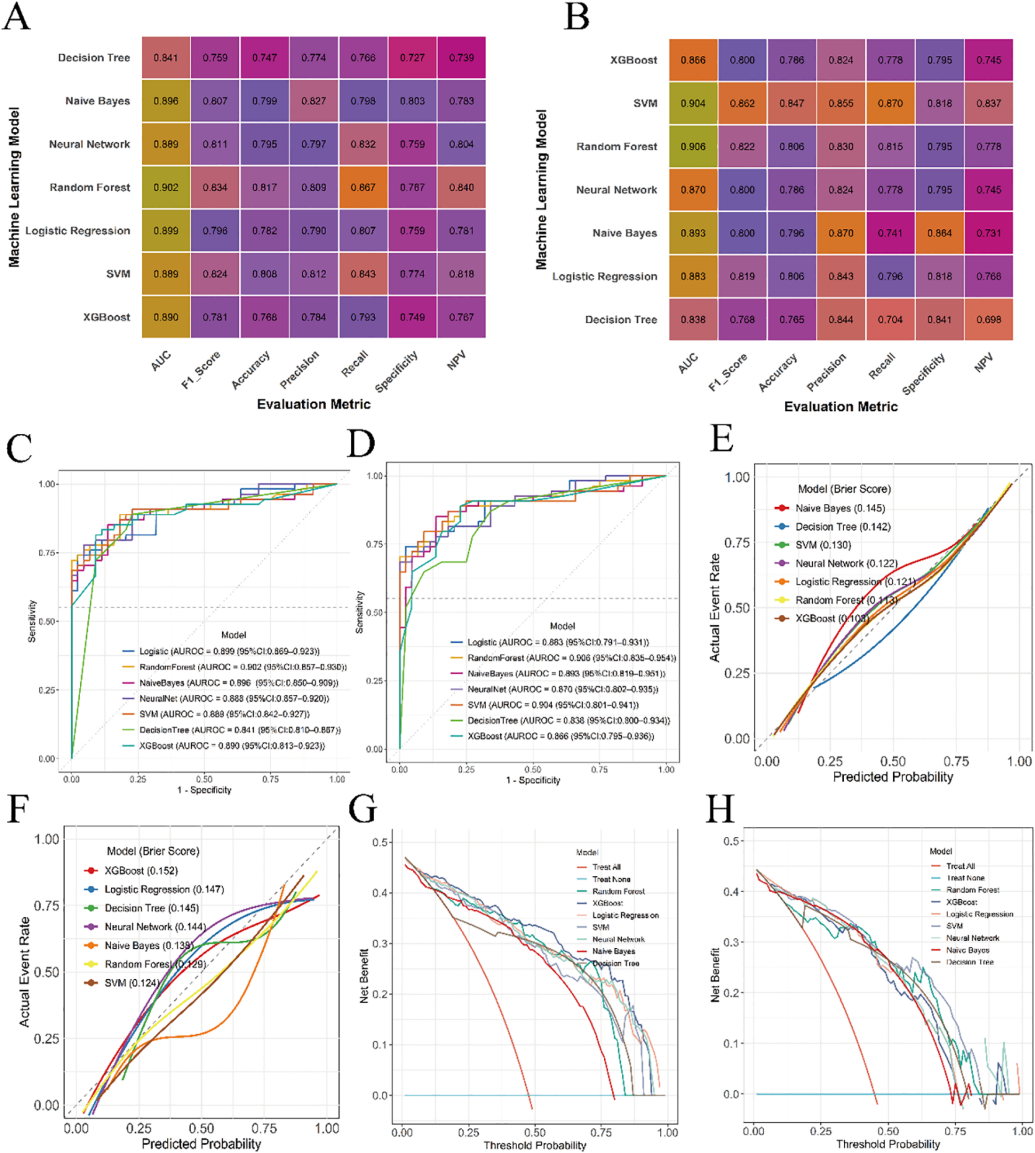
Performance evaluation of seven machine learning models for predicting bone metastasis in newly diagnosed prostate cancer. **(A, B)** Heatmaps of model performance in the training **(A)** and internal validation **(B)** sets. **(C, D)** Receiver operating characteristic (ROC) curves with 95% confidence intervals (CIs) in the training **(C)** and internal validation **(D)** sets. **(E, F)** Calibration curves of the seven models in the training **(E)** and internal validation **(F)** sets. The diagonal dashed line indicates perfect calibration. **(G, H)** Decision curve analysis (DCA) of the seven models in the training **(G)** and internal validation **(H)** sets. “All” and “None” represent the treat-all and treat-none strategies, respectively.

Seven machine learning models were developed and evaluated, including Random Forest (RF), Logistic Regression (LR), Support Vector Machine (SVM), XGBoost, Neural Network (NN), Naïve Bayes (NB), and Decision Tree (DT). Their predictive performances in the training and validation cohorts are summarized in **Figures 2A, B**.

Among all models, the RF model demonstrated the best overall performance. In the training cohort, the RF model achieved an AUC of 0.902 (95% CI: 0.857–0.930), an F1-score of 0.834, an accuracy of 0.817, a precision of 0.809, a recall of 0.867, a specificity of 0.767, and an NPV of 0.840. In the validation cohort, the RF model maintained robust discrimination, yielding an AUC of 0.906 (95% CI: 0.835–0.954), an F1-score of 0.822, an accuracy of 0.806, a precision of 0.830, a recall of 0.815, a specificity of 0.795, and an NPV of 0.778 (**Figures 2A–D**).

Calibration analysis demonstrated good agreement between predicted and observed probabilities. The RF model achieved Brier scores of 0.113 and 0.129 in the training and validation cohorts, respectively, indicating satisfactory calibration performance (**Figures 2E, F**).

Decision curve analysis further demonstrated that the RF model provided consistently higher net clinical benefit than most competing models across a wide range of threshold probabilities in both cohorts (**Figures 2G, H**).

### 3.4 Interpretability of the model

**Figure 3:**
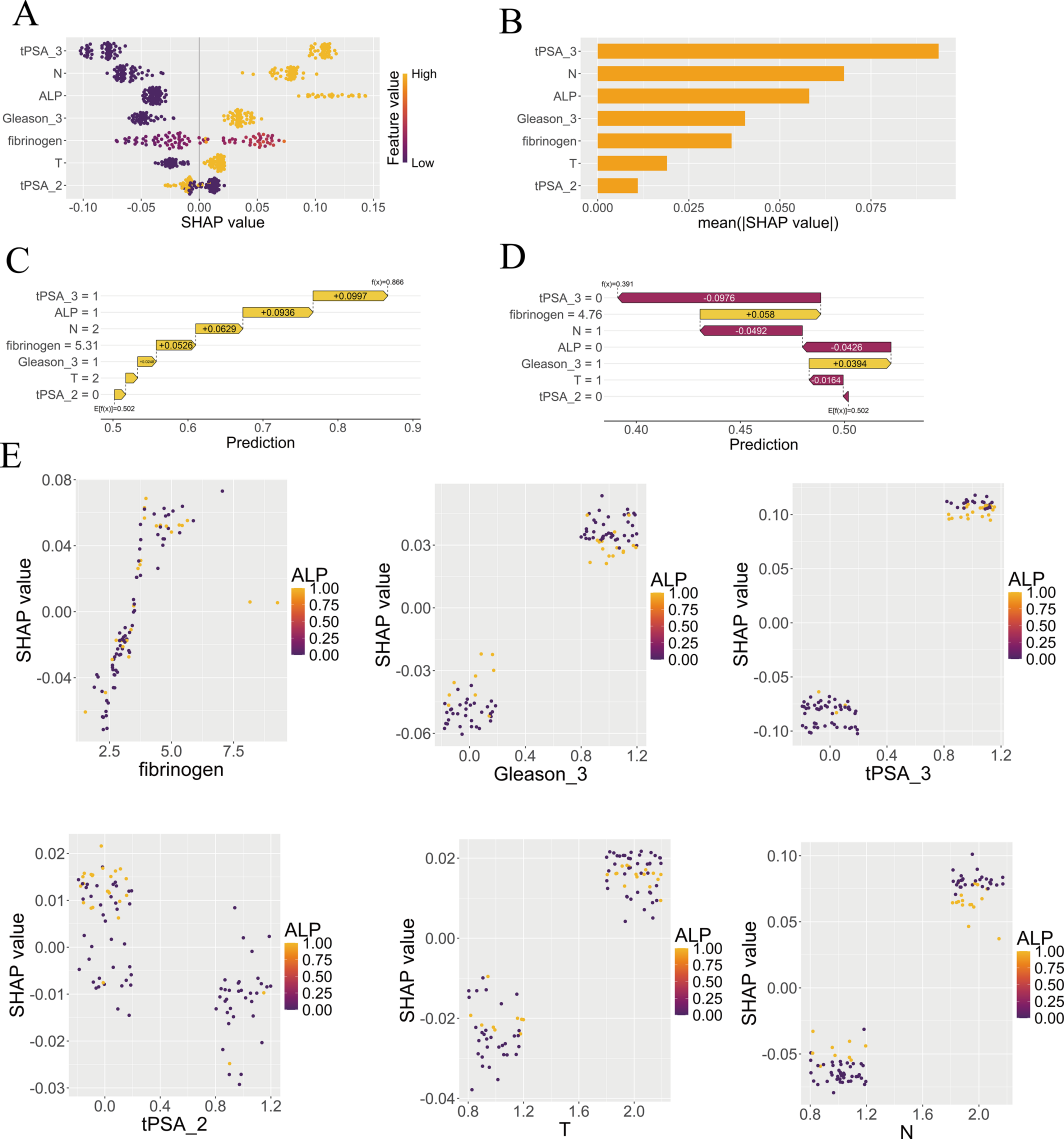
SHAP-based interpretation of the Random Forest model for predicting bone metastasis. **(A)** SHAP summary (beeswarm) plot showing the contribution of each feature to model predictions. **(B)** Feature importance ranking based on mean absolute SHAP values. **(C)** SHAP waterfall plot for a representative high-risk patient. **(D)** SHAP waterfall plot for a representative low-risk patient. **(E)** SHAP dependence plots showing the effects of the six predictors and their interactions with ALP.

The SHAP beeswarm plot quantitatively demonstrates the contribution of each predictive feature to bone metastasis (BM) risk prediction (**Figure 3A**). On the X-axis, positive SHAP values indicate that a feature increases the predicted risk of BM, whereas negative values indicate a reduced risk. Each point represents an individual patient, and the color reflects the feature value, with yellow representing higher values and purple representing lower values. The features are ranked on the Y-axis according to their overall importance. As shown in **Figure 3B**, tPSA ≥100 ng/mL (tPSA_3) was the most influential predictor, followed by regional lymph node metastasis (N stage), ALP >125 U/L, Gleason score >8 (Gleason_3), fibrinogen, clinical T stage, and tPSA 20–100 ng/mL (tPSA_2).

To further illustrate model interpretability at the individual level, SHAP waterfall plots were generated for representative high-risk and low-risk patients (**Figures 3C, D**). In the waterfall plots, E[f(x)] represents the average model output across all samples, whereas f(x) represents the individualized predicted probability of BM based on a specific combination of clinical features. For the high-risk patient (**Figure 3C**), tPSA_3, ALP, N stage, and fibrinogen contributed substantially to an increased predicted probability of BM (84.8%). In contrast, the low-risk patient (**Figure 3D**) exhibited a lower predicted probability (39.1%), reflecting the combined effects of favorable clinical characteristics.

The SHAP dependence plots (**Figure 3E**) further illustrate how individual features influence model predictions. The relationships between feature values and SHAP values are shown for fibrinogen, Gleason_3, tPSA_3, tPSA_2, T stage, and N stage. Point color represents ALP values, allowing visualization of potential interactions between ALP and the other predictors. These plots demonstrate that the effect of each feature on BM risk is not strictly linear and may vary according to the values of interacting variables.

### 3.5 Online web application deployment

**Figure 4:**
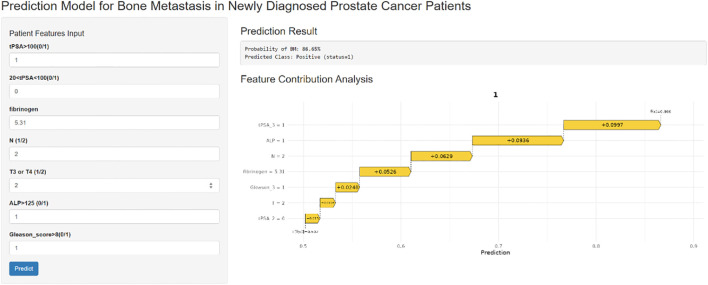
Web-based prediction platform for bone metastasis risk assessment. The interface allows users to input clinical variables and obtain the predicted probability of bone metastasis together with an individualized SHAP-based explanation of feature contributions.

Based on the optimal predictive model (Random Forest), a web-based application was developed for individualized prediction of bone metastasis risk in newly diagnosed prostate cancer patients (https://taoqiu725.shinyapps.io/prostate/). The application incorporates six clinical variables, including total prostate-specific antigen (tPSA), fibrinogen, regional lymph node metastasis status (N stage), clinical T stage, alkaline phosphatase (ALP), and Gleason score. After entering the required variables, users can obtain an individualized probability of bone metastasis. In addition, the application provides a SHAP-based waterfall plot to visualize the contribution of each predictor to the final prediction, thereby enhancing model interpretability and clinical usability. A representative example of the web interface is shown in **Figure 4**. In this case, a patient presenting with multiple high-risk features, including tPSA >100 ng/mL, elevated ALP, lymph node metastasis, advanced T stage, high Gleason score, and increased fibrinogen level, was assigned a predicted bone metastasis risk of 86.6%. The accompanying SHAP waterfall plot illustrates the individualized contribution of each predictor, with tPSA >100 ng/mL and elevated ALP exerting the greatest impact on the final prediction.

## 4 Discussion

Although PSA screening helps in the early detection of prostate cancer, the PSA screening rate is relatively low in underdeveloped regions of China. As a result, the incidence of PCa is significantly lower than in Europe and the United States. Moreover, not all PCa patients have the same bone metastasis risk at the time of diagnosis (2, 9). Consequently, many patients present with bone metastasis at the time of their first diagnosis, which severely impacts their quality of life and survival duration. This may partly explain the relatively high prevalence of bone metastasis observed in our cohort (53.2%), which may reflect both the referral characteristics of tertiary medical centers and the lower PSA-screening rate in our region, resulting in a greater proportion of patients presenting with advanced disease at diagnosis. In this multicenter, retrospective study, we utilized clinical data from two medical institutions to develop and validate an interpretable machine learning model for predicting the risk of prostate cancer bone metastasis. First, clinical data from the training set were analyzed using the Boruta algorithm, which identified tPSA, ALP, regional lymph node metastasis, T-stage, fibrinogen, and Gleason score as predictive factors for bone metastasis in prostate cancer. Based on these factors, we developed and validated seven machine learning models for predicting the risk of bone metastasis. The Random Forest model demonstrated the best predictive performance (AUC: 0.902). Calibration curves and clinical decision curves showed satisfactory accuracy, reliability, and clinical applicability, which were further confirmed in the internal validation set. Finally, SHAP analysis was used to evaluate the interpretability of the best model, and an online web application was deployed for personalized prediction of prostate cancer bone metastasis risk.

This study found that tPSA is a significant risk factor for the occurrence of prostate cancer bone metastasis, which is consistent with previous studies (10, 11). PSA is a single-chain glycoprotein secreted by prostate epithelial cells and is one of the tumor markers for prostate cancer. Research has shown that when PSA > 20 ng/ml, the incidence of bone metastasis exceeds 70%, suggesting that PCa patients with PSA > 20 ng/mL should undergo bone scanning to check for BM (8). Chybowski et al. (12) in a study of 521 newly diagnosed prostate cancer patients in the United States found that the bone metastasis rate was low in patients with low PSA levels. In patients with tPSA < 15 ng/ml, the incidence of bone metastasis was 0%; in patients with PSA levels between 15–20 ng/ml, only one case of bone metastasis occurred. Singh et al. (13), in a retrospective study of 68 newly diagnosed prostate cancer patients, found that when tPSA < 10 ng/ml, the incidence of bone metastasis was 0%, while when tPSA > 100 ng/ml, the incidence of bone metastasis was 100%.

ALP is another risk factor. Previous studies have shown that elevated ALP levels are often associated with a higher risk of bone metastasis (14). Hua Hong et al. (15) suggested that as ALP levels increase, the proportion of patients with bone metastasis increases. For every 50 U/L increase in ALP, the risk of bone metastasis in PCa patients increases by 1.839 times. Kamiya et al. (16) found that elevated ALP levels were significantly associated with the occurrence of prostate cancer bone metastasis in 222 prostate cancer patients. Moslehi et al. (17), in a retrospective analysis of 203 prostate cancer patients, found that ALP is an independent risk factor for bone metastasis, with a higher risk of bone metastasis when ALP > 286 U/L. Wei et al. (18) found that ALP independently predicted the sensitivity (57.1%) and specificity (64.8%) of bone metastasis in prostate cancer. Additionally, Chen et al. (2), using ALP at 120 U/L as the cutoff, combined tPSA, Gleason score, and clinical tumor staging to develop a bone metastasis risk assessment model suitable for the Chinese population. Therefore, ALP, in combination with other independent risk factors, can serve as an important tool for predicting prostate cancer bone metastasis.

Previous studies (9, 19) have shown that compared with N0 patients, those with regional lymph node metastasis are more likely to develop BM. Patients with regional lymph node metastasis usually have high histological tumor grades. Studies have pointed out (20) that LNM is the most important prognostic factor for PCa patients and has been shown to be a critical predictor of PCa BCR-free survival, metastasis-free survival, and overall survival. High PSA levels increase the probability of lymph node invasion, and higher Gleason scores also increase the risk of lymph node metastasis (21). Peng C et al. (10) suggested that pelvic lymph node metastasis could be an independent risk factor for PCa bone metastasis. which aligns with the findings of this study. Therefore, assessing the lymph node status in prostate cancer patients is crucial for staging and determining treatment strategies.

Song Chen et al. (2) divided clinical T-stage (cT) into cT1-cT2 and cT3-cT4, or cT1-cT3 and cT4. Their study indicated that the stratification of cT3-cT4/cT1-cT2 showed a stronger ability to distinguish bone metastasis risk than cT1-cT3 and cT4. Therefore, in this study, cTx was categorized as T1/T2 and T3/T4. Boruta algorithm results demonstrated that a higher cT is an important feature for prostate cancer bone metastasis. Gang Bai et al. (22) confirmed that cTx is an independent risk factor for prostate cancer bone metastasis, with a positive correlation between clinical stage progression and bone metastasis risk. Tsu-Ming Chien et al. (23) found that the risk of bone metastasis in T3/T4 prostate cancer was significantly higher than in T1/T2 (OR = 2.26), suggesting that routine bone scanning should be performed in patients with T3 or higher stage prostate cancer at the time of diagnosis. These findings are consistent with the conclusions of this study. A higher tumor clinical stage usually indicates stronger infiltrative capacity, and when the tumor invades blood vessels, cancer cells can spread to the bone through the circulatory system, eventually establishing metastatic foci.

Gleason score is another feature incorporated into the model. The American Urological Association (AUA) recommends (8) routine bone scanning for Gleason scores ≥ 8. McArthur et al. (24) found that in newly diagnosed prostate cancer patients with tPSA < 20 ng/mL and Gleason score < 8, the cohort study demonstrated a 100% negative predictive value, and bone scanning could be avoided. Studies (10)have shown that Gleason score is an independent risk factor for prostate cancer bone metastasis, with an AUC of 0.882 for predicting bone metastasis, sensitivity of 68.2%, and specificity as high as 98.4% (cutoff value of 7.5). In this study, patients were stratified into three risk categories based on Gleason score (<8, 8, >8). Boruta algorithm results showed that a Gleason score >8 is a risk factor for prostate cancer bone metastasis, which is consistent with the findings of previous studies. However, it should be noted that a Gleason score of 8 was not included, which may be due to the fact that most of the patients in this study were in the advanced stage when diagnosed.

This study also found that fibrinogen is one of the risk factors for prostate cancer bone metastasis. Malignant tumor patients often have hypercoagulable states (25). Chung et al. (26) found that serum fibrinogen levels may be a biomarker for diagnosing and predicting the prognosis of malignancies. Wang et al. (27) found that fibrinogen is independently associated with the severity of prostate cancer. Elevated fibrinogen levels may be related to increased serum PSA, higher pathological grade, clinical stage progression, lymph node metastasis, and distant metastasis. Yu et al. (28), in a retrospective cohort study of 234 patients, pointed out that fibrinogen is an easily obtained, low-cost test indicator that helps predict prostate cancer bone metastasis.

Although conventional bone scintigraphy remains the standard for bone metastasis detection in many resource-limited settings, modern imaging such as prostate-specific membrane antigen (PSMA) PET/CT has demonstrated superior diagnostic accuracy. A prospective head-to-head study reported that ^18^F-PSMA PET/CT achieved 98% sensitivity and 99% specificity for bone metastasis detection in newly diagnosed high-risk prostate cancer, compared with 91% sensitivity for ^18^F-NaF PET/CT (29). A 2025 meta-analysis further confirmed that PSMA PET significantly outperforms bone scan (AUC: 0.95 vs. 0.80) (30). Nevertheless, our model retains clinical utility as a pre-screening triage tool rather than a competitor to advanced imaging. In resource-constrained settings where PSMA PET/CT is unavailable or cost-prohibitive (2), our model helps identify low-risk patients who may safely avoid any imaging, and high-risk patients who warrant immediate advanced imaging. This risk-adapted, stepwise strategy is particularly suitable for clinical settings with varying levels of healthcare resources. Direct comparison with PSMA PET/CT in future multicenter studies will further inform the integration of our model into optimal imaging pathways.

Several Chinese population-based prediction models for prostate cancer bone metastasis have been reported. Xu et al. developed a nomogram with a C-index of 0.929 (31). Jin et al. reported a random forest model achieving an AUC of 0.929, with feature selection highly consistent with our study (32). Song et al. developed a radiomics-based XGBoost model with an AUC of 0.921 (33). The performance of our model (AUC 0.902–0.906) is within the range reported in these studies. Direct head-to-head comparison with existing nomograms and guideline-based strategies in an independent external cohort is warranted to further validate its clinical added value.

Machine learning techniques are often referred to as “black boxes,” and the lack of explanation for the prediction process may lead clinicians to question the generated results (34). Therefore, this study employed SHAP values to perform interpretability analysis on the best model (Random Forest). The global explanation quantified the overall contribution of features to outcome prediction, while local explanations visualized the contribution of specific features to the model output in individual cases through SHAP dependence and waterfall plots, significantly enhancing the credibility of the model’s predictions. Additionally, we developed a publicly accessible web application based on the Random Forest model for personalized predictions of individual cases, which holds significant potential for clinical decision-making.

Mechanistically, the six predictors reflect key steps of prostate cancer bone metastasis. Elevated tPSA promotes osteoblastic metastasis via TGF-β/Smad pathway activation (35); ALP elevation reflects osteoblast activity enhanced by tumor-derived exosomal SNHG1 (36); regional lymph node metastasis indicates hematogenous spread potential, often preceding bone dissemination (37); Gleason score ≥8 defines poorly differentiated tumors with enhanced invasive capacity via IGFBP5/NF-κB signaling (38); advanced T stage (T3/T4) increases circulating tumor cell shedding (39); and fibrinogen contributes to pre-metastatic niche construction by remodeling the extracellular matrix (2). Collectively, these six indicators lend biological plausibility to our predictive model.

However, this study has several limitations. First, it was retrospective in nature, which may have introduced selection bias. In addition, the relatively high prevalence of bone metastasis in our cohort may reflect the referral characteristics of tertiary hospitals and the lower PSA-screening rate in our region. Therefore, caution should be exercised when generalizing the model to populations with a lower metastatic burden. Second, although data were collected from two institutions, all cases were pooled and randomly divided into training and validation cohorts. Therefore, the current study only provides internal validation rather than true external validation using an independent cohort. As a result, the model’s generalizability and applicability across different clinical settings remain uncertain and require confirmation in future multicenter external validation studies. Third, the internal validation set was relatively small, which may have affected the stability of performance estimates. Fourth, prostate size measurements and multiparametric MRI parameters (e.g., PI-RADS score) were not available and therefore excluded, even though these variables are increasingly recognized as valuable prognostic indicators in prostate cancer. Temporal validation of model stability across different time periods and sensitivity analysis using continuous variables remain to be explored in future work.

## 5 Conclusion

In summary, this study identified tPSA, ALP, regional lymph node metastasis, fibrinogen, Gleason score, and cT as significant predictors of bone metastasis in newly diagnosed prostate cancer. Based on these factors, we developed and validated an interpretable Random Forest model that demonstrated excellent discrimination, calibration, and clinical utility. The model has been implemented as an interactive web-based tool, which may assist clinicians in the early detection and individualized management of prostate cancer bone metastases.

## Data Availability

The raw data supporting the conclusions of this article will be made available by the authors, without undue reservation.
